# Gun Possession among American Youth: A Discovery-Based Approach to Understand Gun Violence

**DOI:** 10.1371/journal.pone.0111893

**Published:** 2014-11-05

**Authors:** Kelly V. Ruggles, Sonali Rajan

**Affiliations:** 1 Center for Bioinformatics and Health Informatics, New York University Langone Medical Center, New York, New York, United States of America; 2 Department of Health and Behavior Studies, Teachers College, Columbia University, New York, New York, United States of America; University of Stellenbosch, South Africa

## Abstract

**Objective:**

To apply discovery-based computational methods to nationally representative data from the Centers for Disease Control and Preventions’ Youth Risk Behavior Surveillance System to better understand and visualize the behavioral factors associated with gun possession among adolescent youth.

**Results:**

Our study uncovered the multidimensional nature of gun possession across nearly five million unique data points over a ten year period (2001–2011). Specifically, we automated odds ratio calculations for 55 risk behaviors to assemble a comprehensive table of associations for every behavior combination. Downstream analyses included the hierarchical clustering of risk behaviors based on their association “fingerprint” to 1) visualize and assess which behaviors frequently co-occur and 2) evaluate which risk behaviors are consistently found to be associated with gun possession. From these analyses, we identified more than 40 behavioral factors, including heroin use, using snuff on school property, having been injured in a fight, and having been a victim of sexual violence, that have and continue to be strongly associated with gun possession. Additionally, we identified six behavioral clusters based on association similarities: 1) physical activity and nutrition; 2) disordered eating, suicide and sexual violence; 3) weapon carrying and physical safety; 4) alcohol, marijuana and cigarette use; 5) drug use on school property and 6) overall drug use.

**Conclusions:**

Use of computational methodologies identified multiple risk behaviors, beyond more commonly discussed indicators of poor mental health, that are associated with gun possession among youth. Implications for prevention efforts and future interdisciplinary work applying computational methods to behavioral science data are described.

## Introduction

Gun violence in American schools and communities has and continues to be a serious public health concern. Each year, nearly 3,000 youth are killed and approximately 16,000 are injured by guns [1]. Although an individual must be at least 18 years of age to purchase a rifle or shotgun and at least 21 years of age to purchase a handgun, a subset of American youth still gain access to guns, subsequently increasing their risk of engaging in or being the victim of gun-related violence [2], [3].

There are currently two broad areas of discussion regarding gun violence prevention. The first involves controlling access to firearms by eliminating background check “loopholes”, reducing civilian access to high-capacity weapons, and normalizing safe gun storage practices [4], [5]. The second focuses on reducing the stigma associated with poor mental health and increasing access to mental health services to those who may need such support. Both approaches are important components of the gun violence prevention solution; however, this rhetoric appears to be most prevalent in the wake of mass shootings. Therefore, it is important to note that the work presented here views gun violence prevention in the context of mass shootings as well as with regard to more isolated occurrences of firearm-related violence. Indeed, while mass shootings comprise a devastating proportion of deaths in the US (more than 900 individuals have died in mass shootings since 2006 [6]), over 400,000 individuals are victims of other forms of firearm-related violence annually [7]. Additionally, current research confirms that stricter gun control efforts are effective in curbing gun violence and substantially reducing the number of firearm-related injuries and deaths [8], [9]. However, implementing effective gun control related policy changes are complex and politically difficult to legislate in the short term. Consequently, addressing the mental health “angle” has gained popularity as researchers, clinicians, politicians, and educators seek expeditious methods to reduce the incidence of gun-related violence, particularly among youth. More specifically, and particularly in the context of gun violence prevention following a mass shooting, “mental health” is often conflated with violence and loosely defined as a proxy for individuals who are “in crisis” and/or have a “diagnosable problem” [10]–[12]. While poor mental health is a very real concern and cannot be ignored if gun violence at any level is to effectively be prevented, it is only one piece of a much larger and more complex behavioral puzzle.

We posit that viewing gun violence prevention primarily through the lens of mental health is inadequate in providing us with a complete understanding of the factors that are associated with gun violence among youth. The objective of this study was therefore to apply state-of-the-art statistical methods to data from the Center for Disease Control and Prevention’s (CDC) Youth Risk Behavior Surveillance System (YRBSS), one of the nation’s largest and most comprehensive public health datasets on adolescent behavior, to document the complexity of risk engagement and provide a more comprehensive view of the behavioral factors associated with gun possession among youth. The specific aims of this study are: (a) to present, for the first time, the novel application of data-driven analysis methodologies as feasible techniques for visualizing trends and relationships among all behavioral risk factors assessed via the YRBSS, (b) to analyze the associations between gun possession and other risk behaviors across a ten year period (2001–2011), and (c) to describe the implications of these findings for reshaping the gun violence prevention conversation.

## Study Design and Methodology

### Data Source

We utilized data from the CDC’s YRBSS between 2001 and 2011. At the time of these analyses, the 2013 YRBSS data were not yet available. The YRBSS data were selected because of the large sample size (2001, n = 13,601; 2003, n = 15,214; 2005, n = 13,917; 2007, n = 14,041; 2009, n = 16,410; 2011, n = 15,425), public availability, and generalizability to the broader population. The purpose of the YRBSS is to establish nationwide prevalence of adolescent youth engagement in a range of key risk behaviors, and subsequently utilize these data to inform program development, policy efforts, and research priorities. YRBSS data are collected biennially via a validated instrument comprised of approximately 97 items from adolescent youth in grades 9–12 and from public, private, and parochial high schools across the US [13]. YRBSS data are nationally representative and obtained by the CDC via an extensive three-stage cluster sample design. Through this process, a randomized sample of schools across 16 unique strata covering the entire US is selected. The CDC notes that these strata are determined by both population density and the proportion of both Black and Hispanic youth within that geographic area. The sample is also selected to reflect the varying demography of each state, ensuring that the data are spatially representative. School size is accounted for in this process as well. Within each school, classes from each grade are randomly selected to participate and all youth within each selected classroom are eligible to complete the survey. It should be noted that three states currently do not participate in the YRBSS (Washington, Colorado, and Minnesota). The data are then weighted to match national population proportions, using a weight variable that is based on student sex, race/ethnicity, and grade [14]. The final sample size, comprising approximately 13,500–16,500 youth per year, is subsequently representative of youth from each sex, race/ethnicity group, and high school grade level across the US. Each item is categorical, with the number of response categories ranging from 2–8. Extensive details on the YRBSS methodology are described in detail elsewhere [14]. We obtained IRB approval to conduct this research from Teachers College, Columbia University (protocol #13-277) and New York University Langone Medical Center (study #S13-00722). All patient data were anonymized and de-identified by the CDC prior to download and analysis.

### Data Analysis

YRBSS data were initially downloaded from the CDC’s website into SPSS (version 21.0). These data were converted into tab delimited files and subsequently parsed and cleaned in perl (v5.12.3) and analyzed using Matlab (R2014a). We focused on 55 survey questions from the YRBSS, all of which were asked identically at each time point (2001, 2003, 2005, 2007, 2009, 2011) and whose responses could be categorized into a binary (Yes/No) answer. To simplify our analysis, the categorical data were first converted to a binary value, where responses indicating any engagement (>0 times) were categorized as “Yes” and those youth never engaging in the specific behavior being classified as “No”. Similar binary classifications are utilized by the CDC when reporting risk prevalence estimates [15].

Table S1 lists each of these questions, the corresponding survey question number for each year, the response format, and the associated answer indicating a “No” response (0 Days/Times, Never, No, N/A etc.). Additionally, for each question in which a response of “No” specified engagement in a positive behavior the data were reverse coded to account for engagement in positive versus negative behaviors. Additional details on this process can be found in Table S1. Missing or incomplete data were removed from the data set. In most survey items across the ten-year period, missing data accounted for <5% of the sample. Questions that had more than 10% of responses consistently missing included the following: “*How many times have you attempted suicide in the past 12 months?*”, “*How many days have you ever had at least one drink of alcohol?*”, and “*How often do you wear sunscreen on a sunny day?*”. It should be noted that missing data, particularly regarding sensitive items, are considered normal limitations of self-report research. Other research studies report percentages of missing data on similar items ranging from 3% [16] to 46% [17]. Complete details of missing data are supplied in Table S1. All data were weighted based on a weight variable record for each student included within the YRBSS dataset. This variable takes into account the distribution of students by grade, sex, and race in each survey district in order to match the national population proportions. [13].

### Applying Computational Methods to Risk Behavior Data: Presenting a New Application

To obtain a comprehensive view of adolescent engagement in all risk behaviors, we calculated an odds ratio (OR) for each permutation of the 55 item combinations for a total of 3,025 ORs (55×55) per survey year. It should be noted that ORs are calculated via a 2×2 frequency table, where the frequency of participants exposed to and not exposed to a specific condition are computed alongside the frequency of participants engaging in or not engaging in a specific behavior. A ratio accounting for these frequencies is subsequently calculated (Table 1, and formula below). From this value, the likelihood of a participant engaging in both behavior A and behavior B can readily be determined. Cross tabulation data used in OR calculations for each question and survey year can be found in Table S2

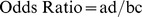
.

**Table 1:** YRBSS Odds Ratio Calculation.

	Frequency of Participants Engaging in Behavior A	Frequency of Participants Not Engaging in Behavior A
Frequency of Participants Engaging in Behavior B	a	b
Frequency of Participants Not Engaging in Behavior B	c	d

The directionality of each OR using raw data is interpreted as follows: given two factors A and B, an OR greater than 1 indicates an increased risk of A (in our example, gun possession) when engagement in or exposure to factor B occurs; while an OR less than 1 indicates a decreased risk of A when engagement in or exposure to factor B occurs. An OR = 1 suggests there is no increased risk of A following engagement in or exposure to factor B. [18].

In order to simultaneously assess associations between all 55 risk behaviors within our sample, we utilized hierarchical clustering, an intuitive visual methodology allowing for the unbiased grouping of adolescent risk behaviors. Hierarchical clustering is a common tool utilized for gene expression analysis, where genes are grouped according to their expression similarity under certain treatment conditions or in different samples types [19], [20]. In our current behavioral science example, each “gene” is instead a survey question, and “gene expression” corresponds to each item’s OR profile. To our knowledge, the methodology described in this study is a novel way to interact with and analyze behavioral survey-based data, and has not been previously used within this context.

**Figure 1:**
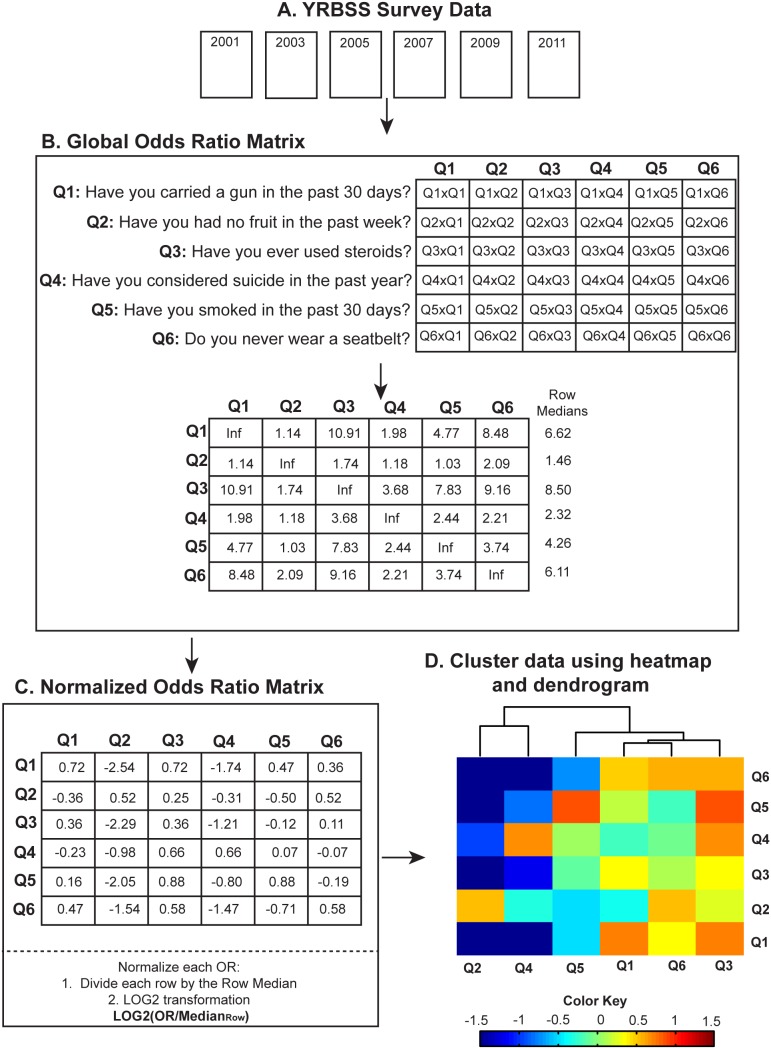
Data analysis framework. (**a**) YRBSS data was processed for each question, including conversion from categorical to binary, data weighting, reverse coding and the removal of missing values. (**b**) Odds ratios (ORs) for each question combination were calculated and stored as a branching odds ratio matrix. ORs comparing the same question (i.e. Q1xQ1, Q2xQ2, etc) are indicated as infinity (Inf) and replaced by the maximum value in the row for all subsequent analysis. (**c**) The global odds ratio matrix was then normalized by dividing by the median value of each row (Row 1 = 6.62, Row2 = 1.46, etc.) and LOG2 transformation. (**D**) The normalized global odds ratio matrix was then used in hierarchical clustering of each risk behavior, and shown as a heatmap (with colors indicating associated normalized odds ratios) and dendrogram (indicating the similarity of each behavior to one another).

Figure 1 provides a simplified example of this methodology using six of the 55 YRBSS survey questions. Following data processing (Figure 1A), we calculated an OR for each question permutation, creating a global OR matrix (Figure 1B). For example, our global OR matrix indicates a strong association between answering “Yes” to Question 3 “*Have you ever used steroids?*” and answering “Yes” to Question 1 “*Have you carried a gun in the past 30 days?*”; with an OR = 10.9 (Figure 1B, Q3XQ1). Instances where the same question is compared to itself (Q1XQ1, Q2XQ2, etc.), the resulting OR is infinity (Inf), and replaced with the maximum non-infinite OR value in each row. The global OR matrix is then normalized by median centering and a log2 transformation (Figure 1C). This normalization aligns the data to a standard normal curve allowing for inter-question comparisons. For example, answering “Yes” to Question 6 “*Do you never wear a seatbelt?*” yielded an OR greater than 1 for every risk behavior permutation calculated. Normalization highlights only those behaviors that have the greatest (Q3 along the Q1 row) or lowest (Q2 along the Q1 row) association within that survey question (Figure 1C). This permits us to focus on behaviors with the largest ORs within each question. Normalized OR values do not speak to the directionality, rather, allow for the relative strength of each association to be compared with one another. This is particularly important when comparing extreme associations within each question, and is required for cross year comparisons. However, for a comprehensive understanding of global risk behavior associations the raw and normalized ORs should be simultaneously evaluated.

This normalized matrix was subsequently used in hierarchical clustering, illustrated as a heatmap and dendrogram, with high normalized ORs in red and low normalized ORs in blue (Figure 1D). The clustering algorithm groups questions with the most similar association profiles in proximity. To create heatmaps and dendrograms we utilized the Matlab *clustergram* function, which performs an agglomerative unsupervised hierarchical clustering using Euclidean distance function and average linkage method to cluster along data columns [19], [20]. In order to validate these clusters, we tested an alternative non-Euclidean distance function (Pearson correlation), and linkage method (complete linkage) using the 2011 YRBSS normalized OR data.

**Figure 2:**
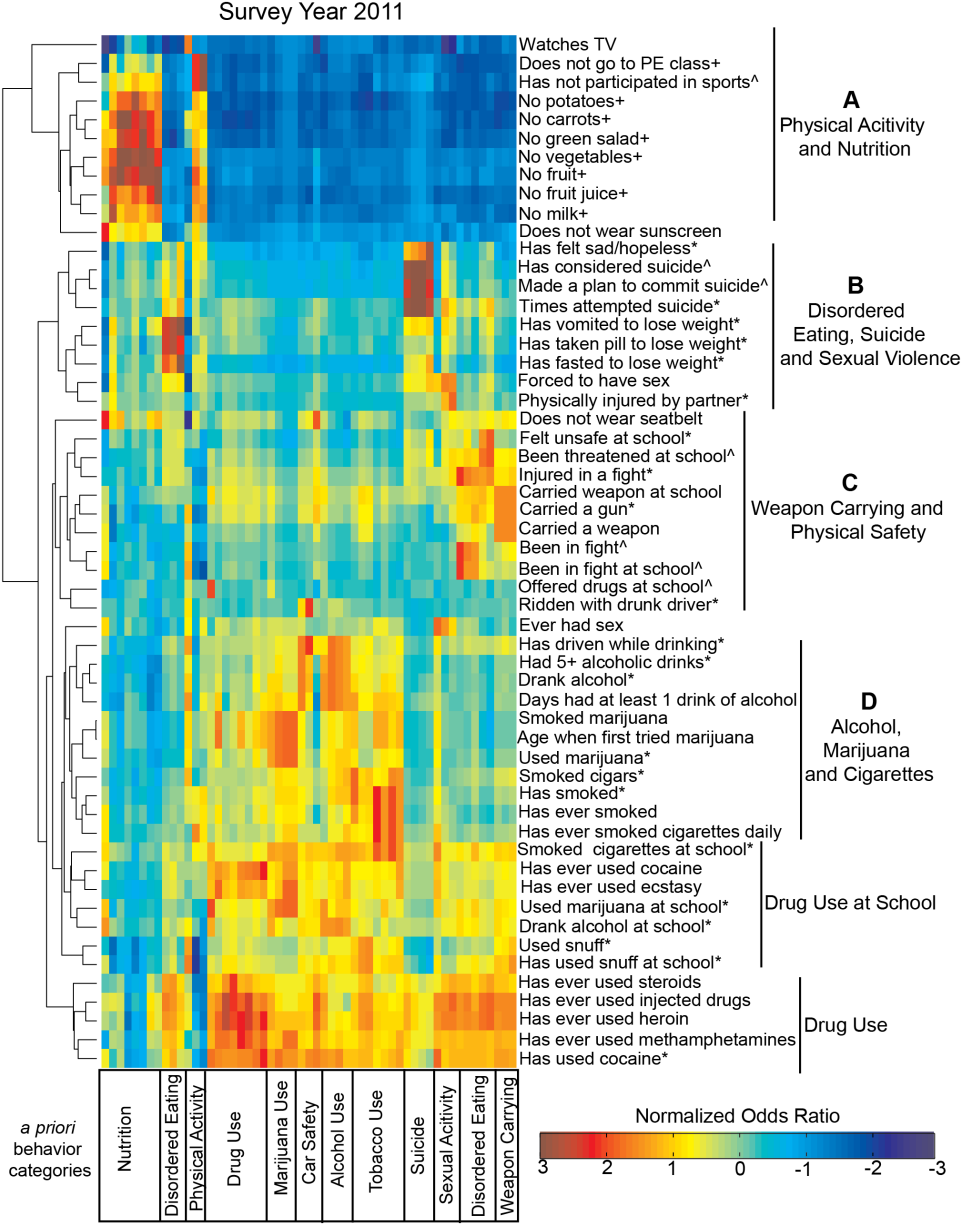
Hierarchical clustering of 2011 comprehensive odds ratios identifies six distinct behavior groupings. Hierarchical clustering, dendogram and heat map based on normalized odds ratios for each permutation of the 55 risk questions. Each row corresponds to questions ordered (1–55), grouped by *a priori* categories listed in order in **Table S1A.** Data was median centered across rows, log2 normalized, clustered along columns and rotated for better visualization (+ in the past week; * in the past month; ∧ in the past year).

**Figure 3:**
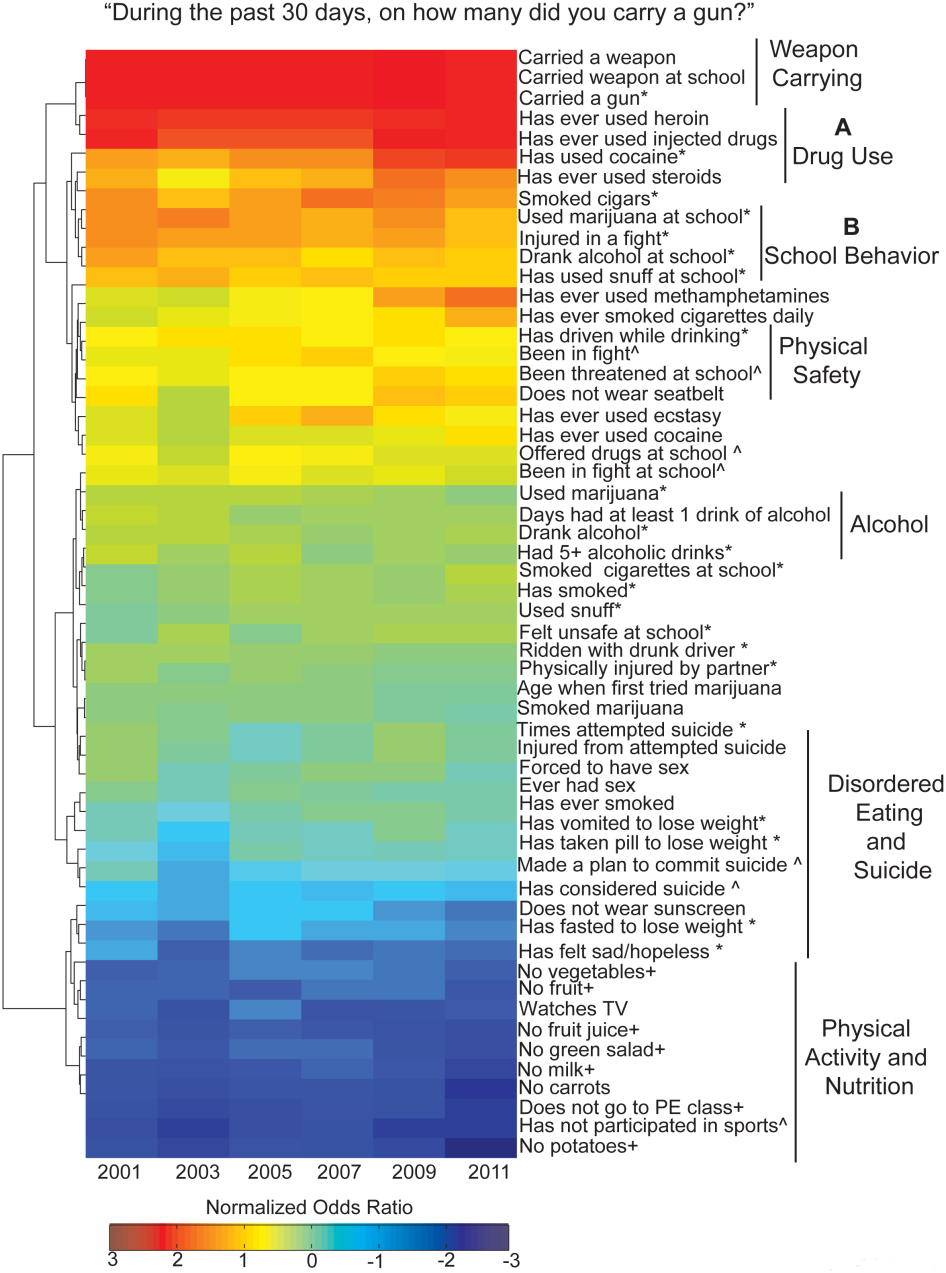
Gun possession odds ratio hierarchical clustering identifies highest and lowest risk behavior associations. Hierarchical clustering, dendrogram and heat map based on odds ratios for weapon carrying and each of the 55 risk questions for each survey year between 2001–2011. Each row corresponds to a survey year. Data was median centered across rows, log2 normalized, clustered along columns and rotated for better visualization (+ in the past week; * in the past month; ∧ in the past year).

In this simple example, not wearing a seatbelt (Q6) and gun carrying (Q1) have similar association profiles, meaning that their highest and lowest ORs correspond with the same behaviors (Figure 1D). Hierarchical clustering is completed for two distinct purposes. The first looks at all OR combinations within each year (Figure 2) where both rows and columns correspond to survey questions (columns match to questions ordered (1–55) in Table S1). The second clusters using OR combinations pertaining to a particular question (Figure 3) across all six time points.

To determine the number of risk behaviors each participant reported engaging in, we considered each of the 55 survey questions, and identified the number of responses indicating engagement in a risk behavior for every participant. The participants were then categorized based on their response to the following item: “*Have you carried a gun in the past 30 days?*” (Yes (indicating >0 instances of gun possession); No (indicating 0 instances). The entire dataset across the ten-year period was considered together and plotted as a box plot. An Analysis of Variance (ANOVA) was subsequently computed to determine if youth reporting recent gun possession engage in significantly more risk behaviors than their peers reporting never carrying a gun.

## Results

### Assessing the Risk Behavior Landscape

Figure 2 illustrates a heatmap and dendrogram that were created following hierarchical clustering analysis on a median centered, log-transformed OR matrix using the 2011 YRBSS dataset (see Table S3 for associated data). This describes a comprehensive assessment of risk relationships; clustering behaviors with similar association fingerprints together and allowing for an unbiased glimpse at similar risk “phenotypes”. Hierarchical clustering algorithms measure the similarity between pairs of observations, in this case how similar the OR patterns were between each survey question (i.e. were the most and least associated risk factors the same for both questions?), and organizes the data by grouping similar elements closer together. [20].

As expected and consistent with previous psychometrics establishing the reliability of the YRBSS instrument [21], items assessing youth engagement in subsets of behaviors formed distinct groups. Specifically, six defined risk behavior clusters were identified by this analysis: 1) physical activity and nutrition; 2) disordered eating, suicide, and sexual violence; 3) weapon carrying and physical safety; 4) alcohol, marijuana and cigarette use; 5) drug use on school property; and 6) overall drug use (Figure 2). Each of these clusters can be understood as having similar associations across all 55 of the risk behaviors assessed. For example, cocaine use in the last month and lifetime methamphetamine use have relatively low associations with behaviors related to poor nutrition (mostly blue in *a priori* nutrition category), and relatively high associations with behaviors in the disordered eating category (mostly orange). The hierarchical clustering algorithm groups these, and other related behaviors (heroin, injected drug, and steroid use) together based on these similarities.

This approach supports the complicated relationship between suicide ideation and disordered eating behaviors [22], [23] (Figure 2B). Our findings also corroborate the strong association between cigarette smoking and marijuana use [24], [25] (Figure 2D). Similarly, this work supports the relationship between increased screen time, lack of participation in physical activity, and poor eating patterns [26], [27] (Figure 2A). Lastly, and in line with our primary study focus, we found that gun possession clustered with risk factors related to physical safety - and in particular, feeling threatened on school grounds (Figure 2C). This latter association indicates that OR patterns across the 55 survey questions concerning physical safety at school are similar to those patterns observed across gun possession; and that these aggressive and violent behaviors are likely symptoms of the same underlying pathology.

Figures created for 2001–2009 yielded comparable clustering results (Figure S1–S5, Table S3). Of particular interest, we found that being offered drugs at school and riding with a drunk driver clustered with weapon possession and engagement in physical violence behaviors in every survey year assessed. Additionally, items evaluating victimization by sexual violence clustered with youth engagement in disordered eating behaviors for two years (2001, 2011) and with weapon possession for the remaining four survey years (2003–2009); together indicating a relationship with both distinct risk behavior subtypes (Figure 2, Figure S1–S5). These findings provide some insight into how youth may assess and estimate risk; and further how a range of risk behaviors may manifest in response to having ever been exposed to violence.

### Associations with Gun Possession among Youth

Though a simultaneous look at all risk behavior relationships considered in the YRBSS is useful, focusing on the multidimensionality of one risk behavior at a time may provide a more valuable context for risk reduction and intervention. For this purpose, we specifically viewed gun possession prevalence trends among adolescent youth across the course of a decade and evaluated the likelihood of engagement in other risk behaviors among those youth also reporting gun possession. This subset of analyses focused on the following YRBSS item: “*During the past 30 days*, *on how many days did you carry a gun?*” The prevalence of youth reporting ever carrying a gun within the past 30 days is as follows: 2001: 5.7%; 2003: 6.1%; 2005: 5.4%; 2007: 5.2%; 2009: 5.9%; 2011: 5.1%. The OR of carrying a gun at least once and engaging in each risk behavior was calculated and subjected to hierarchical clustering as previously described (Figure 3). Since we had access to six time points, we were able to visualize the consistency of these relationships across time. This clustering demonstrated that youth reporting gun possession have the strongest associations with alcohol, tobacco, and other drug use overall and at school across all years studied (Figure 3A, B).

Figure 3 OR values were normalized within each survey year (median centered across years and log transformed as described earlier) to allow for inter-year comparisons. Results illustrated consistent associations with gun possession across the past decade for the majority of survey items (Figure 3, Table S4). Items with inconsistent ORs between years, such as lifetime methamphetamine (raw OR 2001 = 4.7; 2011 = 12.5, normalized OR 2001 = 0.5; 2011 = 1.5) and smoking cigarettes daily (raw OR 2001 = 4.6; 2011 = 9.5, normalized OR 2001 = 0.4; 2011 = 1.1), indicate changing relationships with gun possession. These time-based trends add yet another layer to our risk behavior analysis, requiring further in-depth study to fully appreciate.

**Figure 4:**
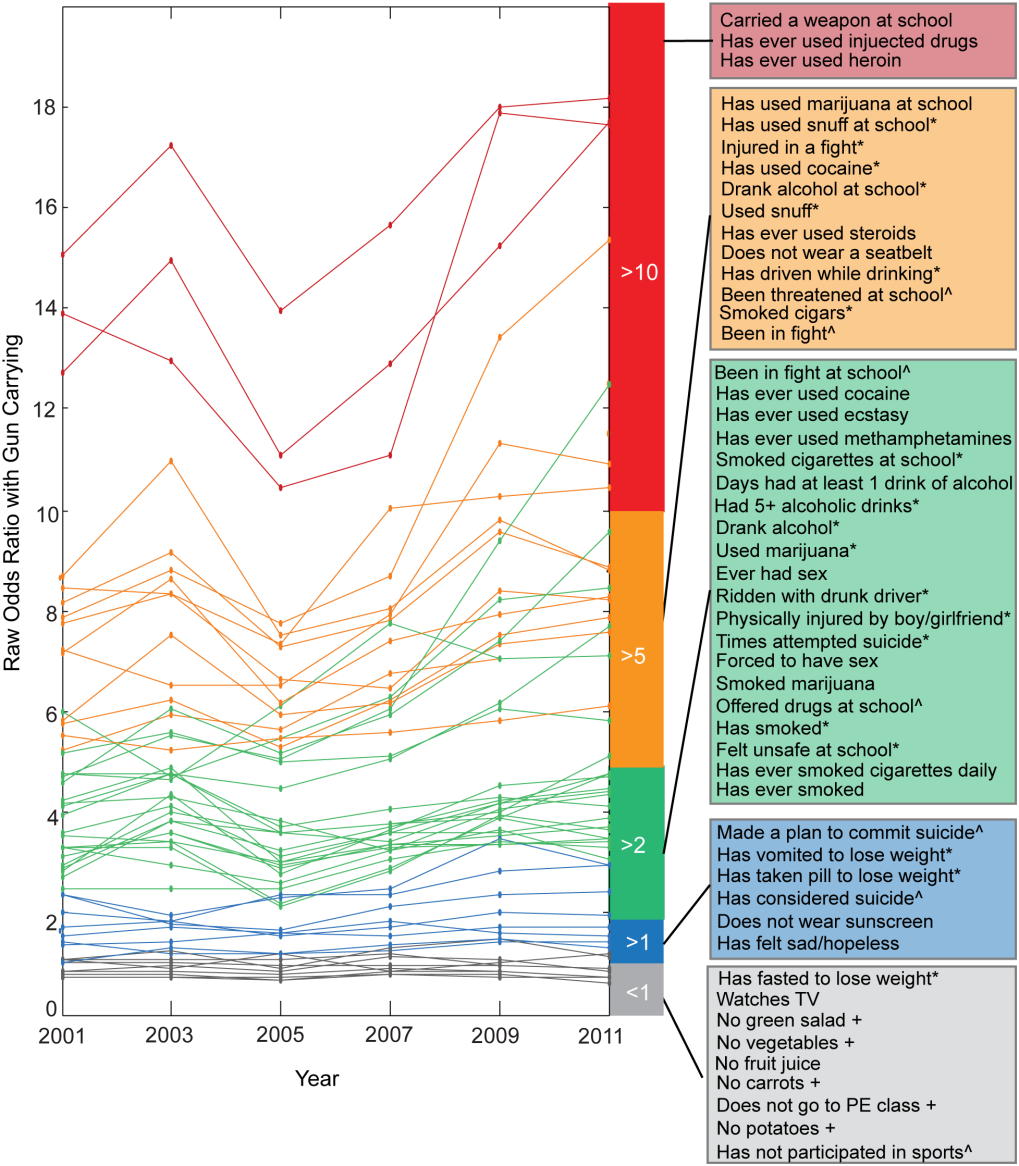
Gun possession is positively associated with the majority of risk behaviors assessed by the YRBSS. Plotting of raw odds ratio (OR) values for 2001, 2003, 2005, 2007 2009 and 2011 for each risk behavior and gun possession, excluding behaviors with infinite OR values (“Carried a weapon”). Behaviors are binned and colored based on their minimum OR across time (>10, red; <5, orange; <2, green; >1, blue; <1, grey). Question labels are indicated in corresponding boxes in order of OR value in 2011 (+ in the past week; * in the past month; ∧ in the past year).

**Figure 5:**
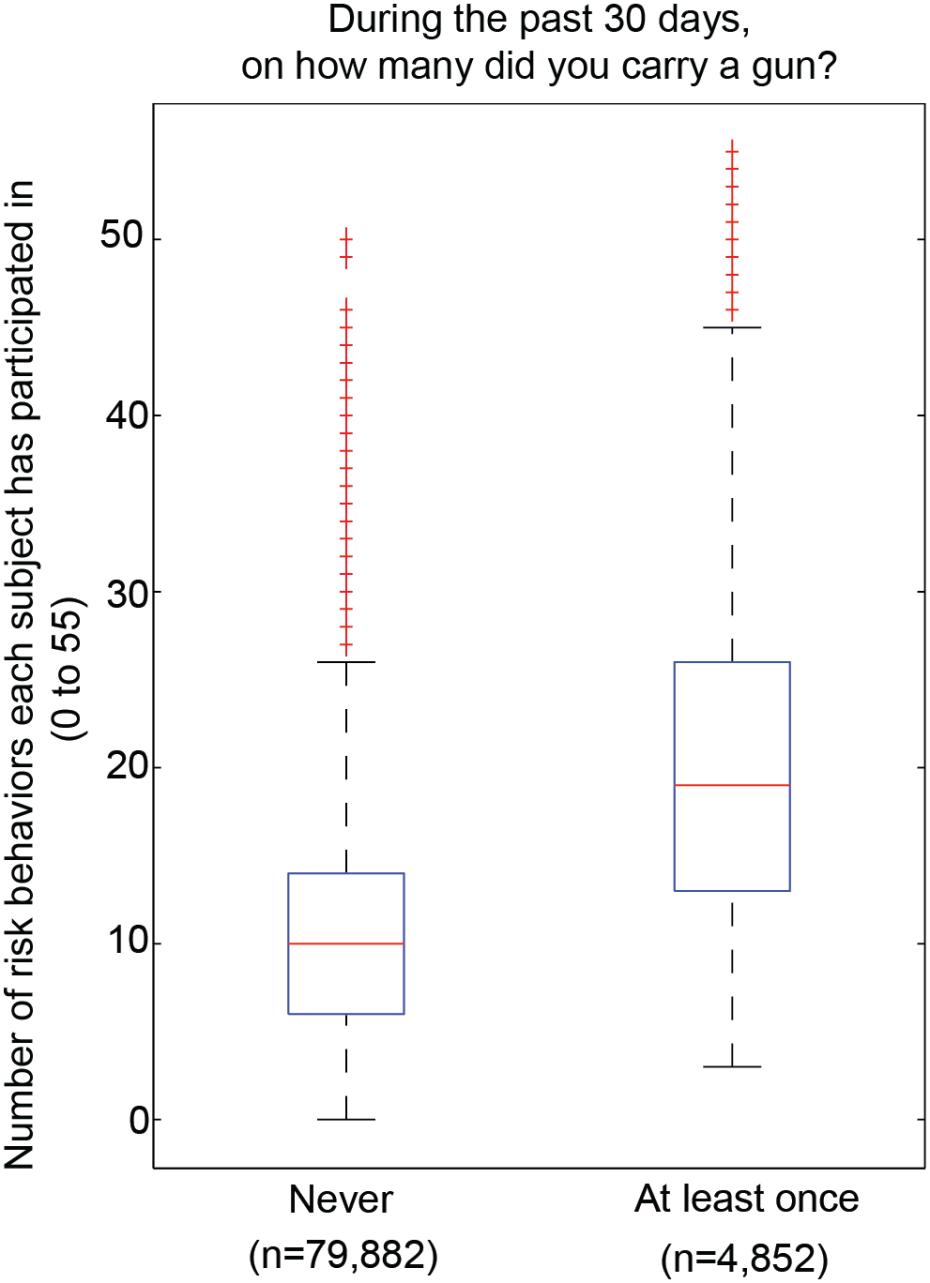
Overall risk behavior participation is increased in subjects reporting gun possession. The number of risk behaviors each subject has participated in was determined by finding the number of positive risk behavior responses for the 55 survey questions considered across all subjects in all years, split by those answering yes to gun carrying versus those who have not carried a gun in the past 30 days. ANOVA comparison between the two groups indicated the difference between groups to be highly significant p<0.000001).

OR normalization is useful for year-to-year comparisons but results in a loss of information regarding overall values of association and directionality of the relationship. For this reason, we also plotted raw OR values between gun possession and each risk behavior and found that the majority of behaviors (43 out of 54) have OR values greater than 1, which have been sustained from 2001–2011 (Figure 4, Table S4). Our findings specifically illustrate that in addition to risk behaviors with the highest associations (alcohol, tobacco, and other drug use; feeling unsafe and being threatened at school), youth reporting gun possession are also more likely to have been the victim of sexual assault, to be engaging in disordered eating behaviors, to not wear sunscreen regularly, and to have recently ridden in a car with a drunk driver (Figure 4). Moreover and upon further examination, our work demonstrates that youth carrying guns are significantly more likely to engage in any number of risk behaviors in comparison to youth not reporting gun possession (Figure 5). More commonly discussed indicators of poor mental health, including suicide ideation and feeling sad or hopeless, were also and unsurprisingly found to be associated with gun possession. However, the strength of these associations in comparison to other risk factors was notably less (Figure 4, Table S4). These collective findings are supported by a growing body of research that indicates that health issues among youth are not isolated concerns and must be treated via synergistic and coordinated programs and policies that look collectively at substance use, violence, poor mental health, sexual risk behaviors, risk of unintentional injuries, and other issues. [28]–[30].

## Discussion

This study analyzed nearly five million unique data points across a ten-year period to better understand the complexity of gun possession behavior among a nationally representative sample of adolescent youth. We used novel computational methods to calculate 18,150 odds ratios, allowing for a simultaneous comparison of all 55 risk behaviors in each survey year and identified six risk behavior clusters, including a cluster that underscored a strong association between weapon carrying and physical safety. Results further demonstrated that more than 40 behavioral factors have and continue to be strongly associated with gun possession. The availability of multiple survey years provided 6 independent replicates of information, which we used to verify each association. To our knowledge, similar methods of high throughput OR calculations and subsequent visualization have not been previously reported.

### Study Limitations

There were limitations to this study that ought to be considered when interpreting these results. First, these data were cross-sectional in nature, making it difficult to account for possible confounders that could affect the study’s findings. Though data were normalized to address this concern, acknowledgment of this limitation is necessary for accurate data interpretation. Second, self-report data collection poses challenges. Participants may select extreme responses at random, underreport their engagement in specific behaviors, or choose to not respond at all [31]. However, given the established reliability and validity of the YRBSS instrument [21] and the consistency of response rates across the ten-year period, we posit that the aforementioned issues of self-report were minimized. Third, as the prevalence of gun possession was relatively low, though still very concerning, (approximate range: 5%–6% of youth reporting recent gun possession per year) we must also account for potential false positives. In order to partially address this, aggregate data from 2001–2011, with more than 4,000 participants reporting gun possession, were also considered (Figure 5).

As noted in the methodology, there are currently three states that do not participate in the YRBSS (Washington, Colorado, and Minnesota). Each of these states varies slightly from one another regarding gun purchase and carrying regulations among adults (ages 18 years and older) [32]. This therefore may influence the ease with which adolescent youth could gain access to guns in each of these three states. However, among the remaining 47 states, there is similar variability in gun regulations. And, as the focus of this present work is on gun possession among adolescent youth, it is important to note that no US state legally allows anyone under the age of 18 to possess firearms of any kind. Further, the extensive YRBSS sampling and weighting procedures, which are designed to account for national population proportions and be representative of youth across the US helps account for the bias associated with these three states’ lack of participation. [21].

Additionally, there are inherent limitations to clustering algorithms that can affect the interpretation of these analyses. Our study relied on an agglomerative (“bottom-up”) clustering approach, which starts with each feature (i.e. behavior) in its own cluster and recursively merges the most similar features together to form larger clusters. Factor proximity and cluster shape are established based on two feature similarity measures, a distance metric (most commonly Euclidean distance) and a linkage method [33]. We acknowledge that there other available hierarchical clustering methods in addition to those used in this study and that the method we chose has associated strengths and limitations. Divisive (“top-down”) hierarchical clustering algorithms, in which all features start as a single cluster that is split according to specific stopping criteria, are also frequently used for hierarchical clustering. These algorithms tend to be more precise at the top of the tree, identifying fewer but larger clusters, while agglomerative algorithms are more precise at the bottom of the tree [34]. Since we were working with a relatively small number of features, we were most interested in finding more, smaller behavior clusters, and therefore used an agglomerative approach.

Further, the distance metric and linkage method used by the algorithm can greatly affect the resulting clusters. Euclidean distance is more sensitive to scaling and fluctuations in the data compared with non-Euclidean distance metrics, such as the Pearson correlation [35]. Additionally, alternative linkage methods affect the intercluster distance, i.e. average linkage determines the distance between clusters based on the average distance between any two cluster features while complete linkage uses the maximum distance between any two behaviors, creating more compact clusters [33]. Therefore, in order to verify our clustering method we re-analyzed the 2011 normalized OR data using the non-Euclidean Pearson correlation distance metric and a complete linkage method. In all cases, we found consistency in identified clusters, regardless of the similarity measures used, particularly in clusters relating to (1) physical activity and nutrition, (2) disordered eating, suicide and sexual violence, and (3) weapon carrying and physical safety (Figure 2 and Figure S6–S8). Further, we independently clustered normalized ORs from 5 other survey years (2001, 2003, 2005, 2007 and 2009) and also found comparable results across years (Figure S1–S5). This reproducibility indicates that, for our purposes, this clustering method provided consistent and repeatable behavior groupings.

Lastly, this study’s results demonstrated that more than 40 behavioral factors have and continue to be strongly associated with gun possession. These factors included substance use, feeling threatened or unsafe, poor mental health, and engagement in disordered eating habits. However, it is important to acknowledge that many of these behaviors could be considered proxies for broader social issues (for example, poverty or disenfranchisement) [36]. Indeed, community- and neighborhood-wide factors have been shown to influence firearm-related violence, which we did not account for in this study [37], [38]. As such, our results should be interpreted with this limitation in mind. However, by identifying specific behaviors, this work provides a sense of the range of possible correlates and areas for specific behavioral intervention and prevention that had not all been identified prior to this study. We also underscore that these results do not imply causality. Instead, use of these computational methods has allowed us to approach a complex social problem that has been highly politicized and fraught with bias, and instead understand which risk behaviors frequently co-occur, without influencing our analyses. Understanding whether one behavior or set of behaviors directly cause another, requires more sophisticated intervention analyses outside the scope of our present study. This exploratory work, however, provides an important foundation for future causal analyses. Future work looking to establish causality should control for socioeconomic status, among other factors, to clearly quantify the role of individual behaviors identified in this study as true possible predictors of gun possession. Additionally, we have provided a novel method via which we have been able to establish clusters of risk behavior engagement, ultimately serving as a discovery-driven model for future analyses.

### Implications for Schools and Communities

Earlier this year and in response to the multiple mass shootings that occurred in 2012, the Obama Administration expanded the list of prevention-based recommendations for making schools and communities safer from gun violence, including (a) placing trained police officers in schools; (b) providing greater mental health support for youth by increasing the number of guidance counselors, psychologists, and social workers; (c) making sure that schools have emergency preparedness plans in place; and (d) encouraging schools to implement evidence-based strategies to cultivate a more nurturing environment [10]. Our analysis of nationally representative data over the course of ten years builds upon these recommendations, illustrating that multiple risk behaviors, beyond more commonly discussed indicators of poor mental health, are associated with gun possession among adolescent youth (Figure 4). Although studies have previously reported on the association of some of these risk behaviors with gun carrying, [39]–[41] their methods limited analyses to a smaller and more focused subset of risk behaviors, thereby not accounting for the full complexity of the behavior landscape. One notable exception is research by Furlong and colleagues, which specifically examined national YRBSS data between 1993–1997 using a sample size of over 40,000 adolescent youth [42]. Their work, an important response to the heightened risk of school-based violence that was taking place at the turn of the century, aimed to identify behavioral correlates of weapon possession in schools. In addition to identifying the prevalence of weapon possession on school property during this time period, they identified a significant, and notable, positive correlation between an increased school risk index (a composite score assessing youth engagement in nine possible risk behaviors occurring on school property) and weapon possession [42]. Twelve years later, our work confirms these relationships between school-based risk behaviors (feeling unsafe at school, substance use on school property) and gun possession, specifically, exists. For example, our analyses identified threats to school safety (Figure 2, Figure 3A) and physical safety (Figure 2C) as having particularly strong associations with gun possession through comprehensive OR comparisons. This suggests that direct actions taken by school staff and administrators in addressing school safety can have an important impact in protecting students by reducing their likelihood of choosing to carry a gun. Conversely, our work also demonstrates that certain risk factors (for example, poor nutrition) are not associated with gun possession among youth. Interestingly, Furlong’s work also demonstrated that a subgroup of youth in their sample frequently carrying weapons to school reported no engagement in the other risk behaviors that were studied [42]. This suggested that weapon possession may be an isolated behavior that is difficult to predict, but perhaps also suggested that a broader risk behavior landscape ought to be considered. As such and through our multidimensional analyses, we have since identified a broader set of behaviors associated with gun possession, not considered in any earlier work, which we suggest be explored in more detail in future research and perhaps considered in the development of future prevention efforts.

Our results provide preliminary evidence that shifting our focus from treating one risk behavior at a time to a more holistic “whole child” approach, may better inform effective future policy. For example, comprehensive school-based efforts encouraging the broader development of positive coping mechanisms, social-emotional skills, and risk assessment and emotion regulation strategies to help youth make thoughtful and less impulsive decisions may together be effective alternatives to more focused and unidimensional intervention efforts that typically address one risk behavior at a time. An emerging body of work has also advocated for this “whole child” treatment strategy [43], which this study’s findings support. Additional intervention-based studies, however, are necessary to determine if the aforementioned comprehensive efforts are indeed reasonable approaches for reducing rates of gun violence among adolescent youth.

### Informatics and Public Policy

The use of these exploratory techniques in studying gun violence is novel and we posit that these data-driven methods can provide a clear and objective picture of the behavioral factors that are associated with gun possession. The recent and growing application of informatics methods to a wide range of fields, including genetics, [44] education, [45] and finance, [46] has demonstrated both the versatility and impact of computational methodology in data visualization, network construction, and data modeling. Further, the growing reliance on the collection and analysis of large datasets by public health practitioners underlines the important societal impact informatics methods can have on health promotion, disease and injury prevention, and policy development.

The use of computationally intensive methods to analyze and visualize nationally representative data on adolescent risk behaviors allowed us to consider many adolescent health issues simultaneously, removing the need for the self-selecting of health priorities. Since each OR determines the statistical relationship between two risk behaviors, our global OR matrix captures the complete set of correlations within the defined variable space. These analyses assume that adolescent engagement in one behavior does not preclude nor exclude them from engaging in any of the other behaviors studied. In essence we treat each behavior as an independent event. By normalizing the ORs across each survey question, we were able to identify risk behaviors that were *most* and *least* associated with the given behavior. For risk behaviors such as gun carrying, in which over 50 of the behaviors assessed had OR greater than 1 across all years, we believe this to be particularly useful because it highlights subsets of adolescent youth who are at the greatest risk for future gun possession and those adolescents who would benefit most from school- and community-based early intervention efforts (Figure 3, 4). Recent calls to action to address issues of gun violence in the US have cited a lack of understanding of characteristics of youth who are more likely to carry (and possibly use) a gun [47], [48]. Without a better understanding of this complicated issue it is difficult to obtain support to develop, implement, and evaluate effective intervention tools or appropriate policy. For example, a recent publication identified engagement in physical fighting as one significant correlate of firearm possession among urban adolescents (n = 689) [49]. Our analysis of the YRBSS data (n_sum 2001–2011_ = 84,734) demonstrate that this relationship exists across the ten-year period (Figure 3), thereby supplementing their findings and ultimately providing stronger rationale for pursuing such lines of research and related prevention efforts.

Additionally, these methods may allow researchers to consider data in novel and engaging ways. This includes the clarity provided by visualizing trends and relationships between and among variables from which one can more easily explore data and draw conclusions. For example, the President’s gun violence prevention proposal only briefly mentioned additional behavioral factors (such as bullying and substance use) [10]. The present study’s findings, however, provide much needed evidence that systematically addressing multiple behavioral factors is crucial to the gun violence prevention solution.

## Conclusions

Efforts to promote health and simultaneously prevent the onset of injury, violence, and disease, are often thwarted by a lack of understanding of factors that contribute to and predict behaviors of interest. Further, public health crises tend to inform research directions. However, establishing research agendas and corresponding hypotheses immediately following a crisis (such as a mass shooting), likely contributes to bias [50]. The present study therefore aims to assess adolescent gun possession using data-driven analysis and publically available data to contribute to this important public health issue. Our study uncovers the multidimensional nature of gun possession, which can inform and improve firearm-related violence prevention, research, and policy efforts.

## Supporting Information

Figure S1 **Hierarchical clustering of 2001 comprehensive odds ratios.** Hierarchical clustering, dendrogram and heat map based on normalized odds ratios for each permutation of the 55 risk questions in 2001. Each row corresponds to questions ordered (1–55), grouped by *a priori* categories listed in order in **Table S1A.** Data was median centered across rows, log2 normalized, clustered along columns and rotated for better visualization (+ in the past week; * in the past month; ∧ in the past year).
(TIF)

Figure S2 **Hierarchical clustering of 2003 comprehensive odds ratios.** Hierarchical clustering, dendrogram and heat map based on normalized odds ratios for each permutation of the 55 risk questions in 2003. Each row corresponds to questions ordered (1–55), grouped by *a priori* categories listed in order in **Table S1A.** Data was median centered across rows, log2 normalized, clustered along columns and rotated for better visualization (+ in the past week; * in the past month; ∧ in the past year).
(TIF)

Figure S3 **Hierarchical clustering of 2005 comprehensive odds ratios.** Hierarchical clustering, dendrogram and heat map based on normalized odds ratios for each permutation of the 55 risk questions in 2005. Each row corresponds to questions ordered (1–55), grouped by *a priori* categories listed in order in **Table S1A.** Data was median centered across rows, log2 normalized, clustered along columns and rotated for better visualization (+ in the past week; * in the past month; ∧ in the past year).
(TIF)

Figure S4 **Hierarchical clustering of 2007 comprehensive odds ratios.** Hierarchical clustering, dendrogram and heat map based on normalized odds ratios for each permutation of the 55 risk questions in 2007. Each row corresponds to questions ordered (1–55), grouped by *a priori* categories, listed in order in **Table S1A.** Data was median centered across rows, log2 normalized, clustered along columns and rotated for better visualization (+ in the past week; * in the past month; ∧ in the past year).
(TIF)

Figure S5 **Hierarchical clustering of 2009 comprehensive odds ratios.** Hierarchical clustering, dendrogram and heat map based on normalized odds ratios for each permutation of the 55 risk questions in 2009. Each row corresponds to questions ordered (1–55), grouped by *a priori* categories, listed in order in **Table S1A.** Data was median centered across rows, log2 normalized, clustered along columns and rotated for better visualization (+ in the past week; * in the past month; ∧ in the past year).
(TIF)

Figure S6 **Hierarchical clustering of 2011 data using a complete linkage method and the Euclidean distance metric.**
(TIF)

Figure S7 **Hierarchical clustering of 2011 data using an average linkage method and the correlation distance metric.**
(TIF)

Figure S8 **Hierarchical clustering of 2011 data using a complete linkage method and the correlation distance metric.**
(TIF)

Table S1 **YRBSS Question Information.** **(A)** Survey question information, including the answer number indicating a “No” response (1 corresponds to A; 2 to B), the survey question number in 2001–2011, whether or not the question was reverse coded, the response format for each question and the % of subjects who did not respond (% missing). **(B)** Response format key worksheet.
(XLSX)

Table S2 **Summary of survey answers for YRBSS 2001–2011.** Cross tabulate count of events for each of the 55 survey questions monitored across all years. (A) number of participants answering yes to both negative risk behaviors, (B) number of participants answering no to both negative risk behaviors, (C) number of participants answering yes to the first survey question and no to the second, and (D) the number of participants answering no to the first survey question and yes to the second.
(XLSX)

Table S3 **YRBSS Odds Ratio Matrices (2001–2011).** Normalized and raw odds ratio matrices for all pairwise variable comparisons for all 55 survey questions for each year.
(XLSX)

Table S4 **YRBSS Odds Ratio Matrices assessing associations with reported gun carrying between 2001–2011.** Normalized and raw odds ratio matrices for all pairwise variable comparisons between “Carried a gun” and each of the 55 other survey questions.
(XLSX)
