# Supplementary figures and images for: Gun Possession among American Youth: A Discovery-Based Approach to Understand Gun Violence
Source: PLoS One. 2014 Nov 5;9(11):e111893. doi: 10.1371/journal.pone.0111893 (PMC4221159; doi:10.1371/journal.pone.0111893)

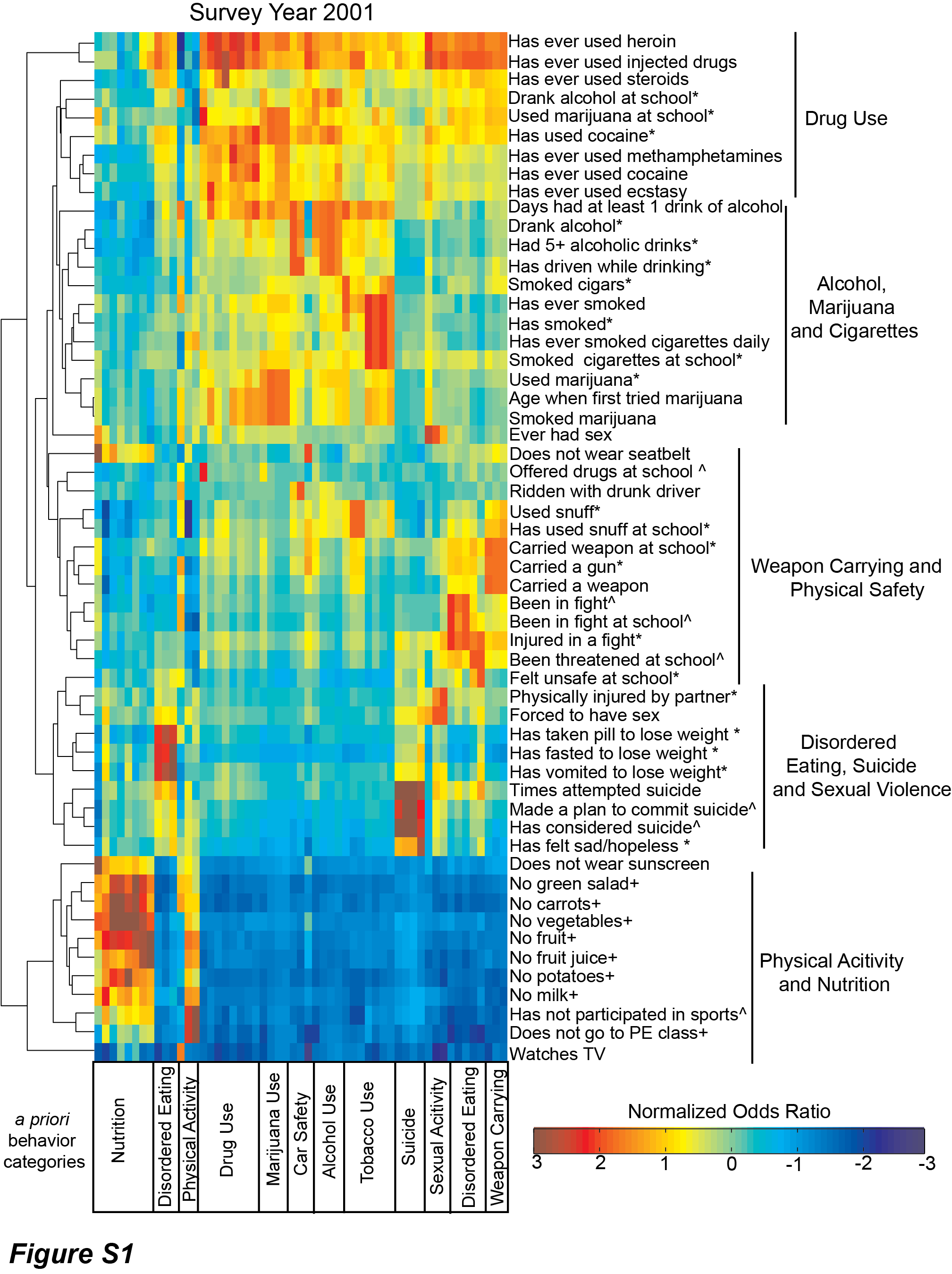

Supplement: Figure S1 — Hierarchical clustering of 2001 comprehensive odds ratios. Hierarchical clustering, dendrogram and heat map based on normalized odds ratios for each permutation of the 55 risk questions in 2001. Each row corresponds to questions ordered (1–55), grouped by a priori categories listed in order in Table S1A. Data was median centered across rows, log2 normalized, clustered along columns and rotated for better visualization (+ in the past week; * in the past month; ∧ in the past year). (TIF) [file pone.0111893.s001.tif]

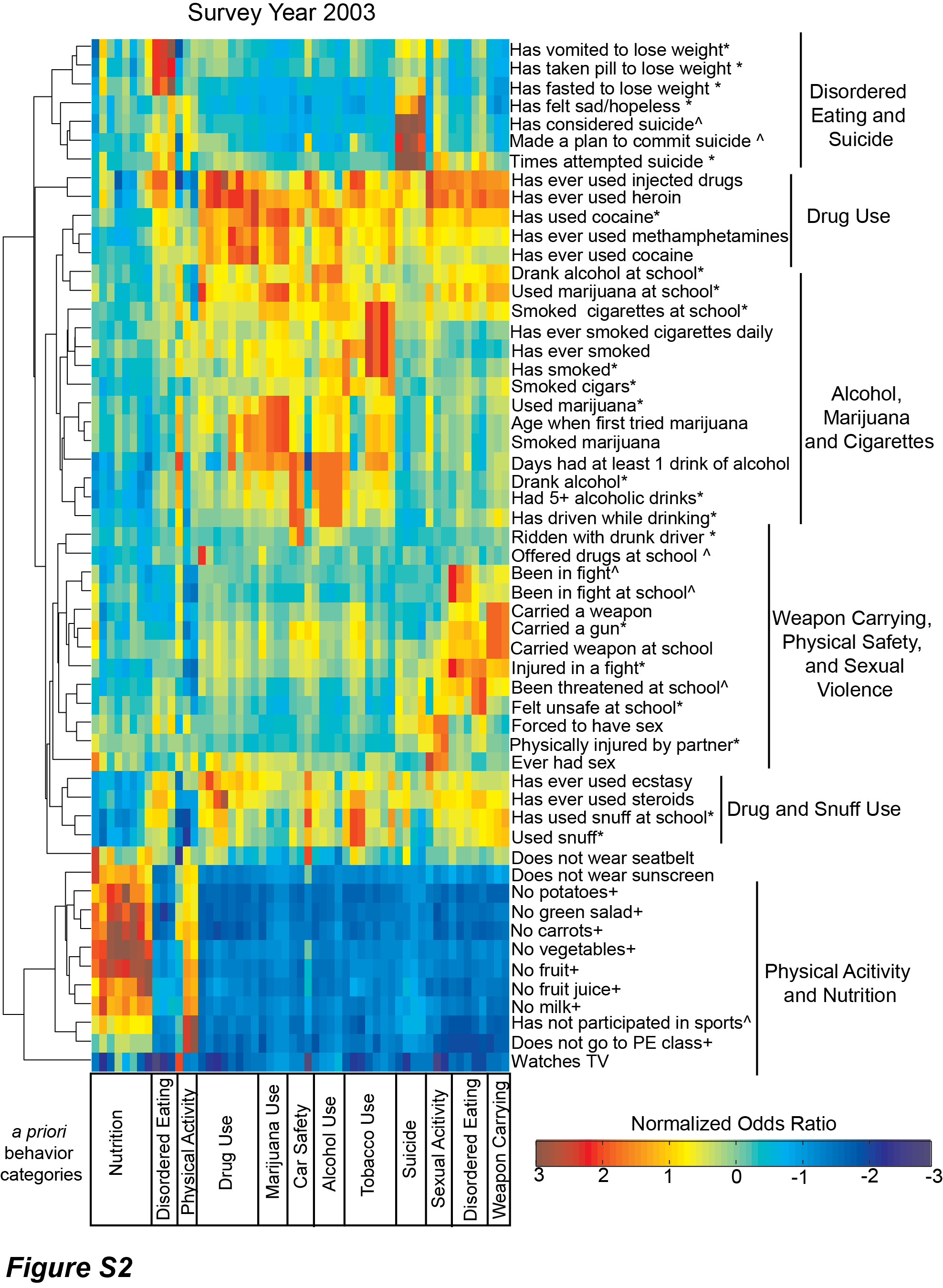

Supplement: Figure S2 — Hierarchical clustering of 2003 comprehensive odds ratios. Hierarchical clustering, dendrogram and heat map based on normalized odds ratios for each permutation of the 55 risk questions in 2003. Each row corresponds to questions ordered (1–55), grouped by a priori categories listed in order in Table S1A. Data was median centered across rows, log2 normalized, clustered along columns and rotated for better visualization (+ in the past week; * in the past month; ∧ in the past year). (TIF) [file pone.0111893.s002.tif]

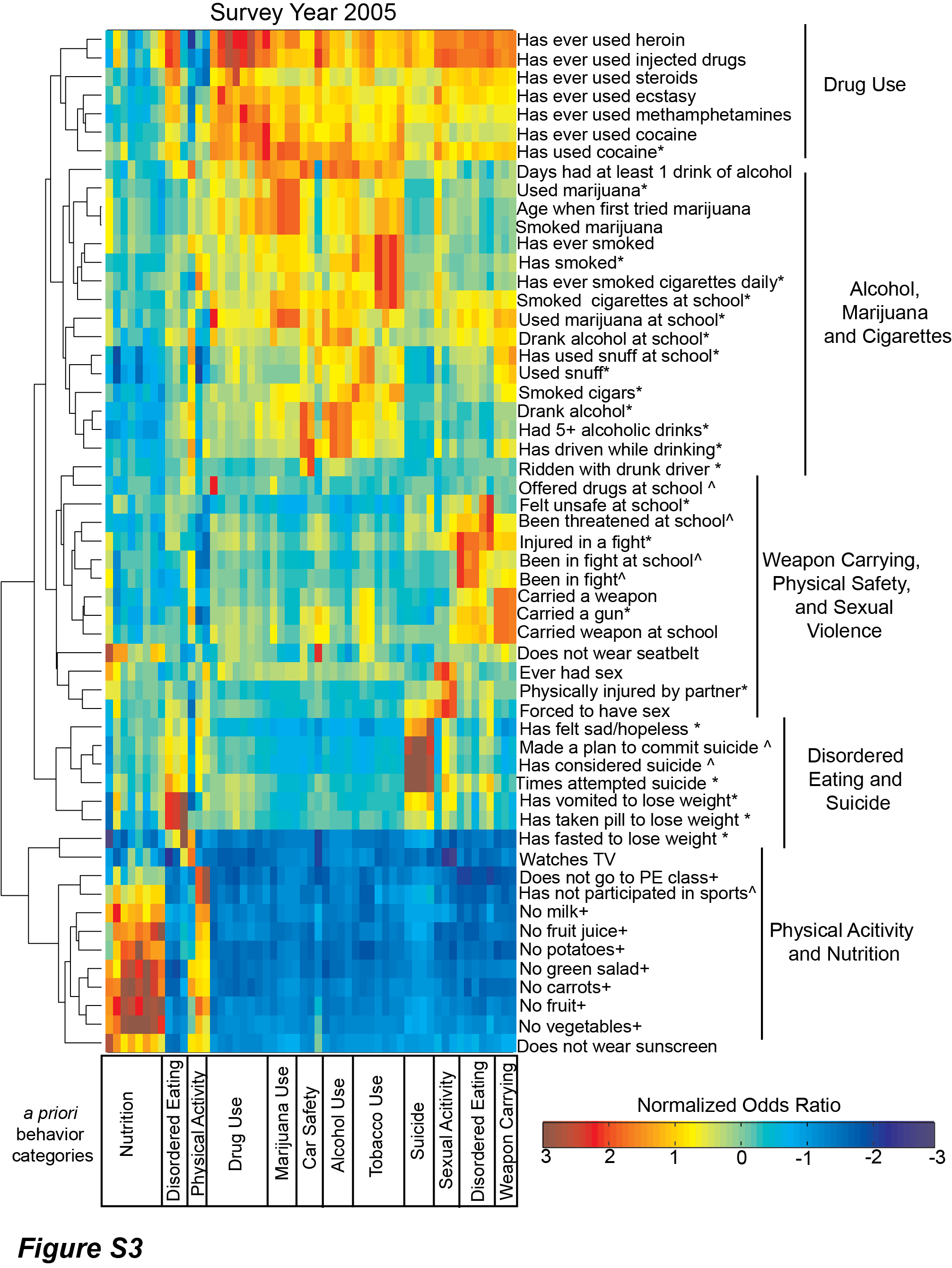

Supplement: Figure S3 — Hierarchical clustering of 2005 comprehensive odds ratios. Hierarchical clustering, dendrogram and heat map based on normalized odds ratios for each permutation of the 55 risk questions in 2005. Each row corresponds to questions ordered (1–55), grouped by a priori categories listed in order in Table S1A. Data was median centered across rows, log2 normalized, clustered along columns and rotated for better visualization (+ in the past week; * in the past month; ∧ in the past year). (TIF) [file pone.0111893.s003.tif]

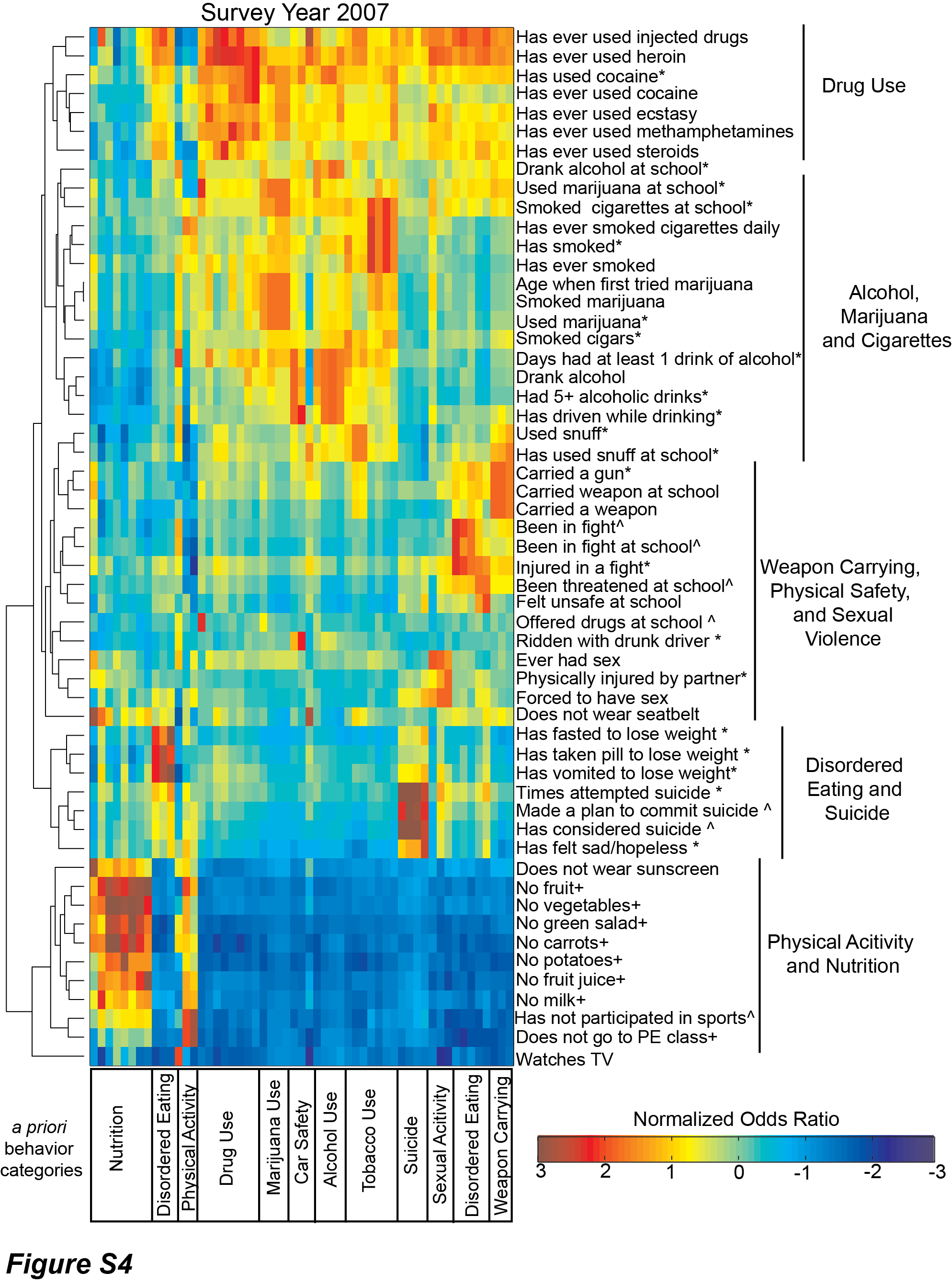

Supplement: Figure S4 — Hierarchical clustering of 2007 comprehensive odds ratios. Hierarchical clustering, dendrogram and heat map based on normalized odds ratios for each permutation of the 55 risk questions in 2007. Each row corresponds to questions ordered (1–55), grouped by a priori categories, listed in order in Table S1A. Data was median centered across rows, log2 normalized, clustered along columns and rotated for better visualization (+ in the past week; * in the past month; ∧ in the past year). (TIF) [file pone.0111893.s004.tif]

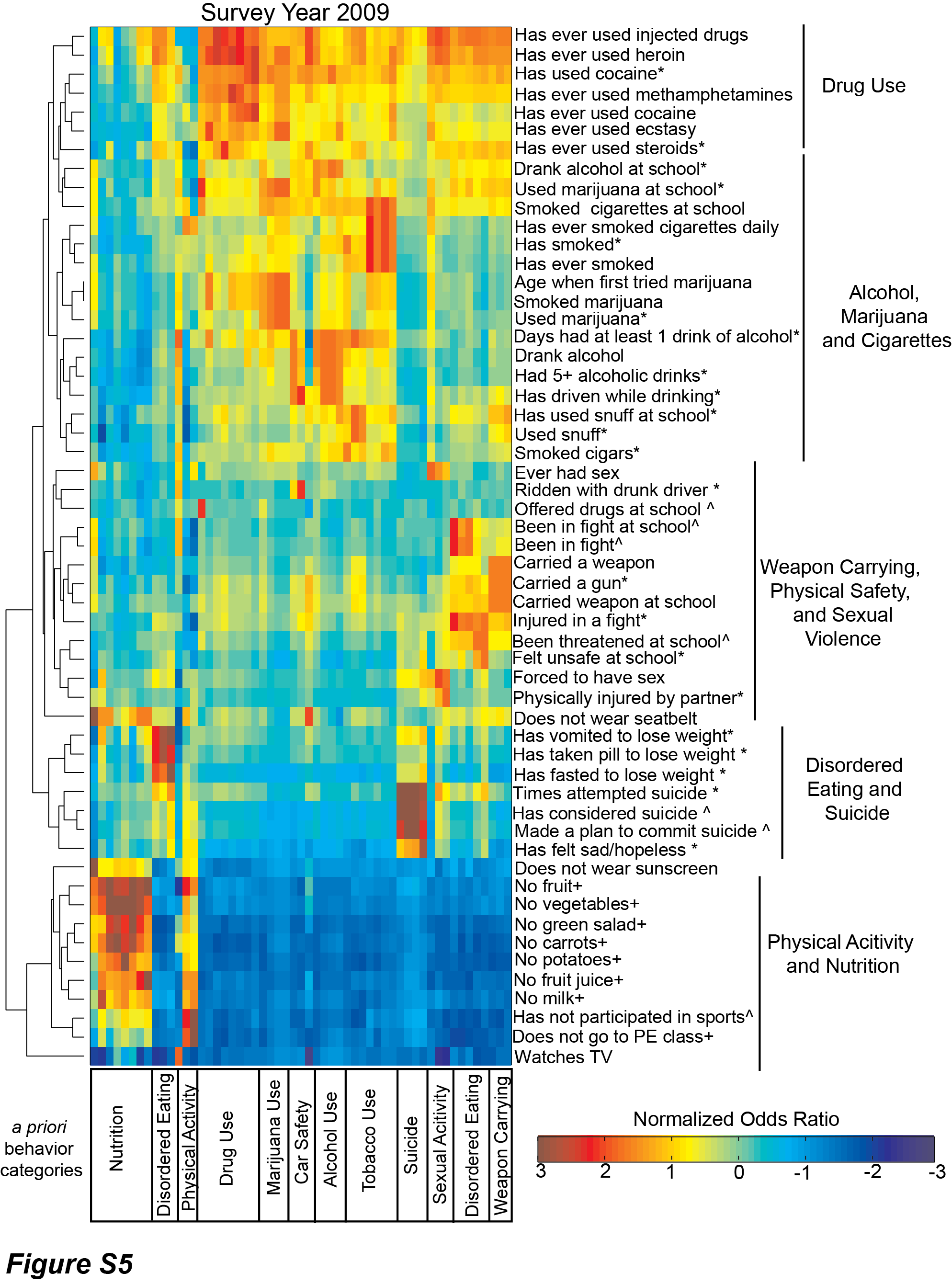

Supplement: Figure S5 — Hierarchical clustering of 2009 comprehensive odds ratios. Hierarchical clustering, dendrogram and heat map based on normalized odds ratios for each permutation of the 55 risk questions in 2009. Each row corresponds to questions ordered (1–55), grouped by a priori categories, listed in order in Table S1A. Data was median centered across rows, log2 normalized, clustered along columns and rotated for better visualization (+ in the past week; * in the past month; ∧ in the past year). (TIF) [file pone.0111893.s005.tif]

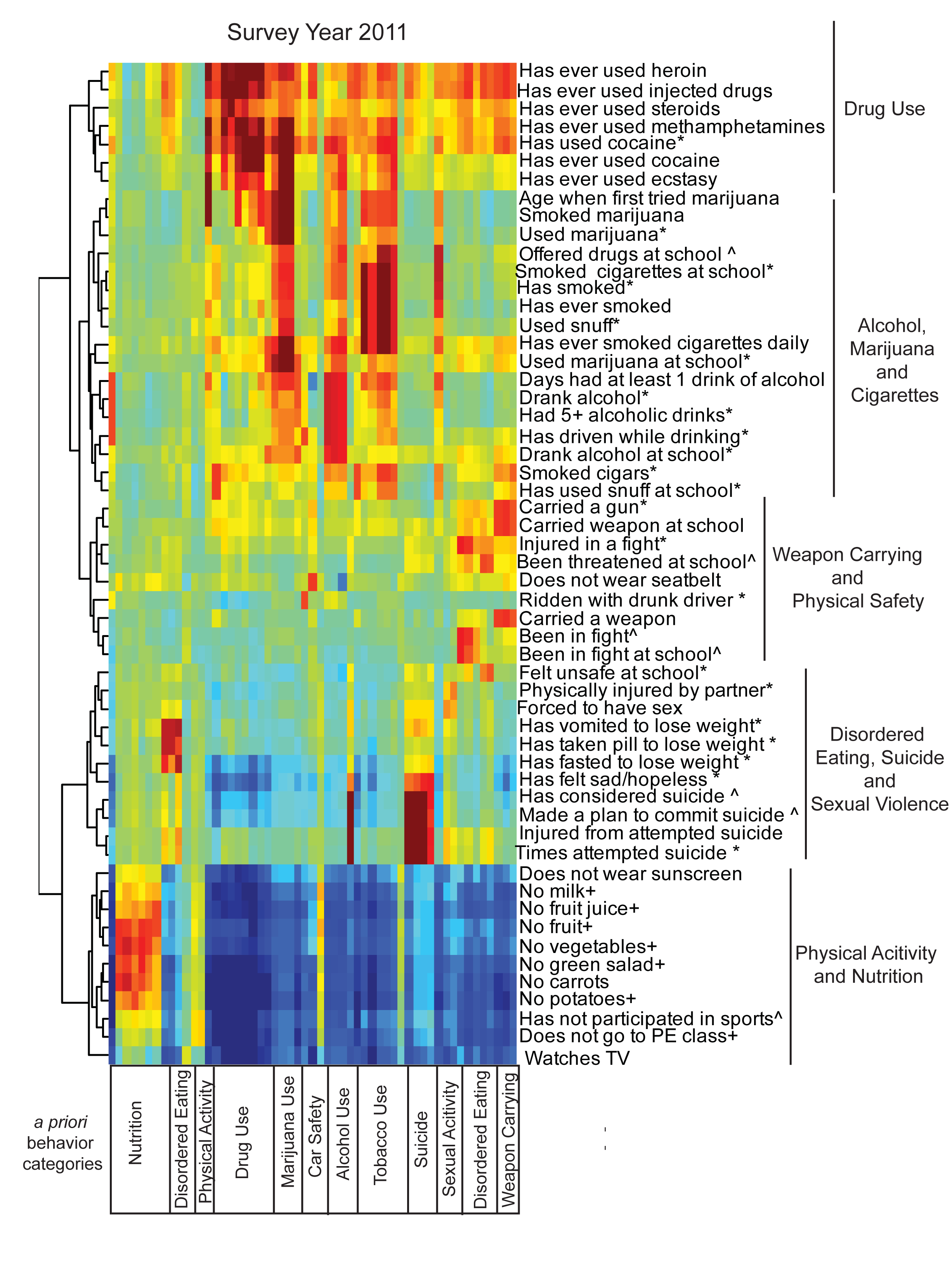

Supplement: Figure S6 — Hierarchical clustering of 2011 data using a complete linkage method and the Euclidean distance metric. (TIF) [file pone.0111893.s006.tif]

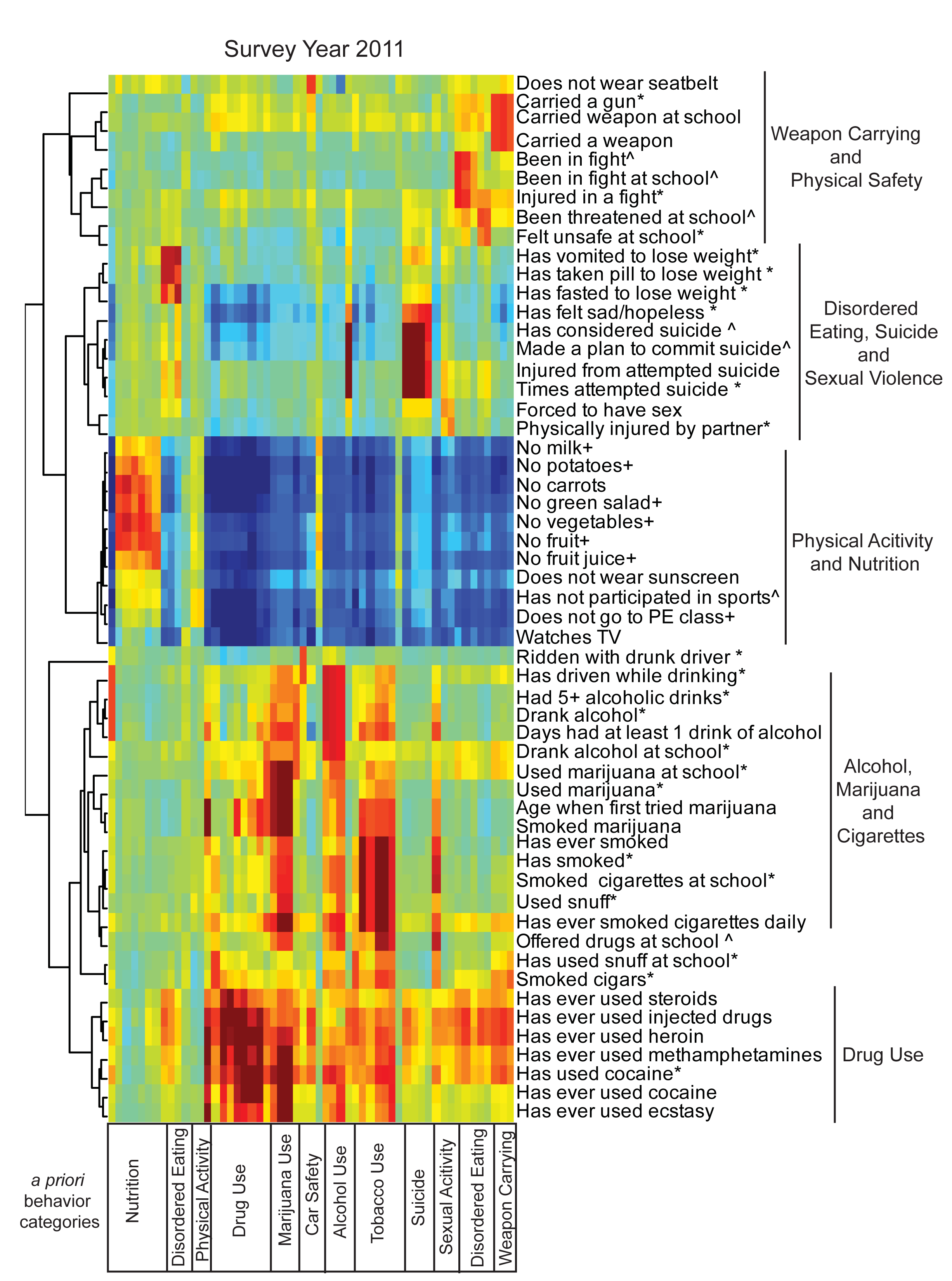

Supplement: Figure S7 — Hierarchical clustering of 2011 data using an average linkage method and the correlation distance metric. (TIF) [file pone.0111893.s007.tif]

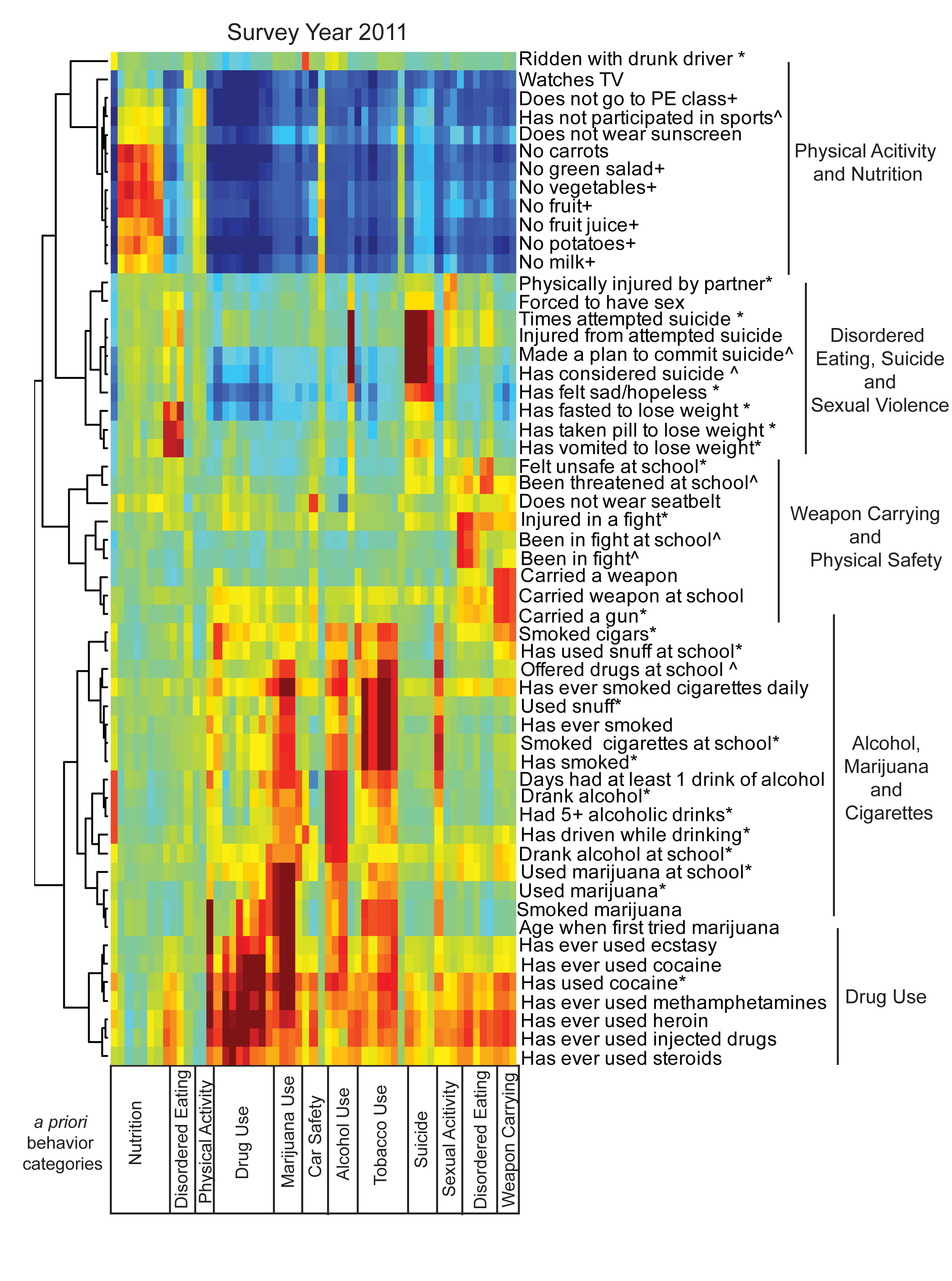

Supplement: Figure S8 — Hierarchical clustering of 2011 data using a complete linkage method and the correlation distance metric. (TIF) [file pone.0111893.s008.tif]
